# National eHealth strategies: a comparative study of nine OECD health systems

**DOI:** 10.1186/s12913-025-12411-7

**Published:** 2025-02-18

**Authors:** Klas Palm, Anders Brantnell, Michael Peolsson, Nurgül Özbek, Gustaf Hedström

**Affiliations:** 1 Department of Industrial Engineering and Management, Uppsala University, Uppsala, Sweden; 2 Swedish eHealth Agency, Stockholm, Sweden; 3 Department of Immunology, Experimental and Clinical Oncology, Genetics and Pathology, Uppsala University, Uppsala, Sweden

**Keywords:** EHealth strategies, Comparative analysis, Health systems, Implementation, Follow-up, Policy evaluation

## Abstract

**Background:**

The development of effective eHealth strategies is critical to enhancing healthcare systems’ efficiency and outcomes. However, there is limited comparative analysis of eHealth strategies across health systems, particularly in terms of their vision, objectives, implementation methods, and follow-up processes. This study compares the eHealth strategies of nine health systems, focusing on three key dimensions: vision and objectives, means to achieve objectives, and structures for follow-up.

**Methods:**

A comparative qualitative analysis was conducted using publicly available eHealth strategy documents from nine health systems: Australia, Denmark, Estonia, Finland, Norway, Sweden, the UK (NHS England), Catalonia (Spain), and the USA (Veterans Affairs). The analysis mapped these systems’ visions, objectives, implementation methods, and follow-up structures.

**Results:**

Findings show that most systems articulate clear visions and strategic goals. However, there is considerable variability in the level of detail regarding the means of achieving objectives and structures for follow-up. Australia and Estonia present the most comprehensive strategies, with clear tasks, responsibilities, timelines, and follow-up mechanisms. In contrast, countries like Sweden and Catalonia provide less detailed strategic plans, particularly in terms of follow-up processes.

**Conclusions:**

While most studied health systems include clear visions and strategic goals, there is variability in the detail and comprehensiveness of their implementation and evaluation frameworks. Strategies with detailed implementation plans and follow-up processes, such as those from Australia and Estonia, offer valuable models. Further research is recommended to explore the practical impact of these strategies on healthcare delivery, patient outcomes, and system efficiency. Additionally, the role of stakeholder involvement in shaping these strategies warrants further investigation.

**Supplementary Information:**

The online version contains supplementary material available at 10.1186/s12913-025-12411-7.

## Background

The World Health Organization (WHO) defines eHealth as “the cost-effective and secure use of information and communications technologies in support of health and health-related fields, including health-care services, health surveillance, health literature, and health education, knowledge, and research” [1]. eHealth offers several promising benefits, including enhanced public health, improved quality of care, and increased productivity and efficiency at both organizational and societal levels [2–6]. With challenges such as an aging population, growing demand for healthcare services, and rising costs, eHealth has emerged as a potential solution to address these issues while achieving broader health objectives [7–9]. For instance, in Europe, the European Union (EU) has integrated eHealth into its health policy to encourage its adoption and development among member states [10–12]. Similarly, several OECD countries, as well as their health systems, including independent healthcare regions, are investing in eHealth initiatives [13]. Additionally, more than 120 WHO member countries, including many low- and middle-income nations, are developing eHealth strategies [14].

Despite its potential, eHealth implementations have often faced significant challenges, with high failure rates reported in both developed and developing countries [15, 16]. In many cases, eHealth landscapes have evolved into fragmented patchworks, making interoperability, development, and management difficult [17]. As a result, there is increasing interest in identifying the factors that influence successful eHealth implementation [18, 19]. A recent review on eHealth policy highlighted several critical factors, including stakeholder involvement, resource availability, follow-up mechanisms, and the clarity of the eHealth strategy itself [19]. A well-defined eHealth strategy can provide a roadmap encompassing visions, objectives, implementation guidelines, and evaluation methods, serving as a driving force for successful eHealth adoption [20, 21].

Strategies can be defined in various ways, ranging from broad to narrow in scope, and may include multiple components. Johnson and Scholes [22] provide a broad definition, stating that “a strategy indicates the direction and scope of an organization over the long term: which achieves advantage for the organization through its configuration of resources within a changing environment, to meet the needs of markets and to fulfill stakeholders’ expectations.” Alternatively, Ginter et al. [23] offer a narrower perspective, describing strategic planning as a set of processes to identify an organization’s desired future and develop guidelines for decision-making to achieve that future. Similarly, the WHO National eHealth Strategy Toolkit emphasizes three main strategic components: a National eHealth Vision, a National eHealth Action Plan, and National eHealth Monitoring and Evaluation mechanisms [24]. Campbell et al. [25] further argue that a strategy fundamentally consists of a vision (i.e., an aspirational statement outlining long-term desired outcomes), objectives (i.e., measurable milestones to achieve the vision), and the means to achieve those objectives. Additionally, Hall and Roussel [26] underscore the importance of evaluation and follow-up as integral elements of any strategy.

Drawing from these definitions, we define an eHealth strategy in this paper as: a vision for how digital health technologies will enhance health outcomes, combined with specific objectives to guide progress, identification of the means (e.g., resources, policies, and tools) necessary to achieve these objectives, and the establishment of structures for follow-up to ensure accountability, evaluation, and continuous improvement.

To develop high-quality eHealth strategies and enhance productivity and efficiency in healthcare systems, it is essential to analyze and learn from existing national eHealth strategies [18, 27]. Therefore, the objective of this paper is to compare commonalities and differences in eHealth strategies across three key dimensions: a) vision and objectives, b) means to achieve objectives, and c) structures for follow-up.

## Method

### Study design

The study employed a comparative qualitative design, guided by the framework proposed by Eisenhardt [28], to analyze eHealth strategies as articulated in existing policies and supporting documentation.

### Data and data collection

To address the study’s objective, we conducted a systematic search to identify appropriate health systems at the national or subnational levels, including regional and local levels. The selection criteria for the health systems included the following: (1) a well-established healthcare and regulatory system, (2) extensive experience in digital transformation, and (3) availability of an eHealth strategy in one of the researchers’ working languages: English, Swedish, Danish, or Norwegian. Additionally, we aimed to include health systems that were comparable in terms of funding mechanisms and healthcare coverage.

An initial search was conducted in late 2022 using the Google search engine, applying a combination of search expressions: *nationally AND strategy AND eHealth/e-health* and *national AND strategy AND digital AND health*. This search yielded 12 potential candidates for further analysis: Australia, Denmark, Estonia, Finland, Germany, the Netherlands, Norway, Sweden, Switzerland, the UK (NHS England), Catalonia (Spain), and Veterans Affairs (VA) in the USA. In early 2023, an in-depth search was performed on the official websites of the respective national and subnational ministries, agencies, and organizations in the fields of health and digitization. Based on the scope and content of publicly available eHealth strategies, the final selection was narrowed to nine health systems: Australia, Denmark, Estonia, Finland, Norway, Sweden, the UK (NHS England), Catalonia (Spain), and the USA (VA).

To ensure the inclusion of the latest strategies and supporting documents, a follow-up search was conducted in late 2024. This follow-up revealed new documents published by Norway and Finland, which were subsequently incorporated into the analysis. All the analyzed health systems are located in OECD countries, characterized by similarities such as being single-payer, tax-funded, and providing universal healthcare coverage. However, the USA stands out due to its mixed system of public and private payers, as well as the lack of universal healthcare coverage. Therefore, we focused on the Veterans Affairs (VA) system in the USA, which offers universal coverage for eligible veterans and is tax-funded, aligning more closely with the other systems in this study. Similarly, Spain is divided into 17 autonomous regions, each with authority over healthcare governance and strategic planning, including the creation of strategic documents. Catalonia was selected due to its independent management of healthcare and because it met our inclusion criteria. NHS England, in turn, is the body responsible for overseeing the National Health Service (NHS) in England, with the primary goal of managing and coordinating healthcare services across the country. It should be noted that, in addition to NHS England, there are three other NHS administrations: NHS Scotland, NHS Wales, and NHS Northern Ireland, which are not included in this study. Given this variation in the study population, all are called health systems.

These nine health systems have been actively involved in eHealth initiatives for over 20 years, providing valuable insights and experiences from the evolution of their eHealth strategies. Table 1 offers an overview of the selected systems and Appendix 1 provides additional details on the healthcare systems of each country. A total of 25 documents were collected for this analysis, including eHealth strategies and related documents from top national level or equivalent (see Appendix 2 for details).

**Table 1:** Overview of health systems and the timeframe of eHealth initiatives in the nine health systems studied

Country/region	Scope	Universally covered & tax financed	Administrative structure	Time frame of eHealth initiatives in the country
Australia	National	Yes	Decentralized	30 years or more
Catalonia (Spain)	Subnational/Regional	Yes	Relatively centralized	20–30 years
Denmark	National	Yes	Decentralized	30 years or more
Estonia	National	Yes	Relatively centralized	20 years
Finland	National	Yes	Decentralized	30 years or more
Norway	National	Yes	Decentralized	30 years or more
Sweden	National	Yes	Decentralized	30 years or more
UK (NHS England)	National	Yes	Relatively centralized	20–30 years
USA (VA)	Subnational	Yes	Relatively centralized	30 years or more

Centralized administrative structure refers to the existence of a body with a larger mandate over health care, while decentralized structures are often made up of several more or less independent bodies (regions/units) responsible for health care

### Data analysis

A comparative text analysis, following the content analysis framework outlined by Elo & Kyngäs [29], was conducted in two parts. First, we mapped the specific eHealth strategies of the nine health systems and analyzed their (1) vision and objectives, (2) means to achieve the objectives, and (3) structures for follow-up. Second, we identified commonalities and differences between the nine health systems in terms of these aspects, adhering to a comparative approach [28]. To ensure credibility, all documents were independently analyzed by two authors, with consensus reached through group discussions.

To analyze the vision and objectives, we focused on what the eHealth strategies explicitly outlined. For the means to reach the objectives, we built on the WHO toolkit and existing research on eHealth policy [19, 24]. First, we examined the development process of the eHealth strategies, acknowledging that strategies grounded in the specific health system context and its challenges are more likely to succeed [19, 24]. Second, we explored the relationship between eHealth strategies and other relevant national strategies, such as those focused on health, digital transformation, cybersecurity, or data governance. Ensuring alignment with these strategies helps avoid conflicts, overlaps, and resource inefficiencies, fostering a more cohesive policy environment [24]. Third, we assessed the coordination of eHealth strategies with other national strategies, as effective coordination is critical for implementing successful digital health policies [24]. Fourth, we analyzed the working methods, including the defined roles and activities for implementing the strategy, since clear responsibilities and actions are fundamental to achieving the objectives [24]. Finally, we considered stakeholder cooperation in implementation of the strategies, as including relevant stakeholders is essential for achieving successful outcomes [19, 24]. These five aspects were crucial for understanding the means to achieve the objectives, and the empirical material allowed us to capture this information effectively.

For analyzing the structures for follow-up, we focused on the descriptions of follow-up and evaluation provided in the obtained documentation. Additionally, the empirical documents were analyzed to determine the extent to which the eHealth strategies offer guidance on vision, objectives, means to achieve the objectives, and structures for follow-up. This analysis formed the basis for evaluating the “quality” of the strategies.

## Results

### Visions and objectives

#### eHealth visions

Although only some of the nine health systems explicitly define an eHealth vision (Australia, Estonia, Finland, Norway, Sweden), all the health systems include a strategy to achieve their eHealth goals. These strategies often feature statements that resemble visions. For instance, Australia’s ultimate vision is to achieve “an inclusive, sustainable and healthier future for all Australians through a connected and digitally enabled health system” [30]. Finland’s vision is focused on “Building a digital foundation for healthcare and social welfare services” [31], while Denmark’s strategy states that “the aim is for patients to experience the health system as a coherent and trustworthy health network for all that is both inherently digital and inherently personal” [32].

Australia, Denmark, Estonia, Finland, Norway, and NHS England all prioritize enhancing health and social services through digital solutions, with a strong emphasis on integration, safety, security, and innovation. In contrast, Sweden and the VA place their respective countries or organizations at the center of their vision alongside IT solutions. Sweden aims to become the world leader in leveraging digitization and eHealth opportunities by 2025, while the VA envisions its coordinating authority, the Office of Information and Technology, as the best IT organization in the government, offering a seamless and secure experience.

In Catalonia, while there is no explicit overarching vision for their digital health strategy, the title of the strategy document, “Building a Digital Health Strategy for Catalonia Together,” reflects the general idea. Notably, this strategy emphasizes participatory governance of eHealth as a key component, distinguishing it from other strategies analyzed.

#### eHealth objectives

All health systems identify eHealth objectives within their eHealth strategies. Based on these objectives, seven broader strategic areas were identified, as summarized in Table 2. These areas align with the overarching goals of improving healthcare efficiency, accessibility, and quality through eHealth initiatives. For example, the Danish National eHealth strategy emphasizes that there is no real alternative to increasing digital cooperation in achieving national health objectives. Although Sweden and the VA state their recognition of the potential for more effective data and information management, it remains unclear in their strategic documents whether they view this as essential for meeting current challenges.

**Table 2:** Strategic areas related to objectives in the nine eHealth strategies

	**Australia**	**Catalonia**	**Denmark**	**Estonia**	**Finland**	**Norway**	**Sweden**	**NHS England**	**Veterans Affairs**
**Strategic Area**									
Patient Involvement	Yes	Yes	Yes	Yes	Yes	Yes	Yes	Yes	Yes
Telemedicine	Yes	Yes	Yes	Yes	Yes	Yes	No	Yes	Yes
Preventive Healthcare	Yes	Yes	Yes	Yes	Yes	Yes	No	No	Yes
Information Management	Yes	Yes	Yes	Yes	Yes	Yes	Yes	Yes	Yes
Standards	Yes	Yes	Yes	Yes	Yes	Yes	Yes	Yes	Yes
Consistent use of Terms	Yes	Yes	Yes	Yes	Yes	Yes	Yes	No	Yes
Data Analysis	Yes	Yes	No	Yes	Yes	Yes	No	Yes	Yes

### Means to reach the objectives

#### The development process of eHealth strategies

In general, the process of developing and updating the strategy is poorly described in available documents. For some health systems, such as Denmark and Sweden, the description is almost completely absent. For the remaining health systems, the process is briefly described. However, it is still possible to distinguish certain similarities and differences. Several health systems describe similar methods, including conducting needs analyses based on patient and care professions, employing forums, workshops, webcasts, town hall meetings, and extensive surveys to identify key themes that form the foundation of eHealth strategies. Almost all health systems except Sweden describe a collaborative process including healthcare providers, patients, technology companies, and government agencies in the strategy development process, but it is not always explicitly documented to provide insights into the extent of cooperation. Health systems such as Australia, Estonia, Finland, Catalonia, NHS England, and VA all describe the use of such methods. However, the extent of the number of people involved in these activities differs across health systems. The two strategies that provide a number are VA, indicating that approximately 150 people participated in various activities, Finland stating that approximately 6,000 people were included (of which approximately 4,500 responded to a survey). Another common approach is to include committees made up of individuals with different skills in the process of developing the strategy.

#### Relation to other relevant national strategies

All nine health systems have eHealth strategies anchored at the highest levels of political governance and linked to national authorities responsible for implementing overarching ambitions at a national or large-scale level. Most of the examined strategies explicitly state that the eHealth strategy serves as a strategic realization of broader national health and social care policies, as exemplified by Australia, Denmark, Estonia, Finland, Norway, and the VA. In some instances, the eHealth strategies are also aligned with other national frameworks, such as those addressing general digitalization, healthcare, research, or the digital economy. For example, Australia and, to a lesser extent, Finland, integrate their eHealth strategies within these broader domains. However, the connection between the eHealth strategy and other national strategies is less clearly articulated in Catalonia and NHS England and is entirely absent in Denmark and Sweden.

#### Coordination of eHealth strategies

In terms of coordinating eHealth strategies at the national level, many health systems, including Australia, Denmark, Estonia, Norway, Sweden, and the VA, have assigned overall coordination responsibilities to a central agency or steering committee, typically operating under their respective ministries of health. In contrast, NHS England has adopted a decentralized approach, delegating this responsibility to local health systems. These local systems are tasked with formulating their own plans to implement the commitments set forth in the NHS Long-Term Plan.

#### Working methods

The working methods described in the eHealth strategies of the analyzed health systems vary significantly in detail and scope, allowing them to be grouped into three distinct levels. Level one: strategies at this level do not provide a clear description of how implementation will be carried out. The VA strategy is the sole example in this category.

Level two: strategies at this level outline the responsibilities for certain measures required to achieve the goals but do not provide detailed descriptions of the methods or processes for implementation. This category includes the strategies of Denmark, Catalonia, Finland, Norway, and Sweden. Level three: strategies in this category identify specific tasks and provide detailed plans for implementation. Examples include those from Australia, Estonia, and NHS England. For instance, Australia’s strategy roadmap clearly identifies the responsible organization, assigns tasks, and specifies the expected timeline for completion.

#### Stakeholder cooperation in implementation

The extent of stakeholder cooperation described in the implementation of eHealth strategies varied among the studied health systems. Several systems—Australia, Catalonia, Estonia, Norway, and NHS England—explicitly highlight the importance of stakeholder collaboration in their strategies. NHS England, however, frames stakeholder cooperation more ambiguously, presenting it as an explicit goal for future development.

In contrast, Denmark, Finland, Sweden, and the VA also reference stakeholder cooperation in their strategic documents, but the information provided is insufficient to evaluate the extent or practical implementation of this collaboration.

### Structures for follow-up

All studied health systems include a follow-up and evaluation process to assess the outcomes of their eHealth strategies, but the level of clarity in describing these processes varies significantly. Catalonia, Sweden, NHS England, and the VA lack explicitly defined follow-up measures in their strategic documents. In contrast, the remaining systems incorporate frameworks where each strategic priority is tied to evaluation measures conducted on a regular basis. Some systems provide particularly detailed and structured follow-up processes. For example, Norway’s national council model with continuous monitoring, evaluation and possible adjustment of the strategy. In support documents for the strategy, there are also clearly defined target values ​​to evaluate efforts against, such as, for example, that the use of certain services at Helsenorge should increase by 20% by the year 2025. Finland also outlines follow-up measures, though some of these remain broadly defined. Catalonia, despite not providing specific follow-up details in its strategic framework, conducts continuous evaluation of eHealth solutions. This evaluation is supported by AQuAS, a public authority under the Ministry of Health of Catalonia, which collaborates with hospitals to generate evidence-based insights.

To summarize, the analysis of eHealth strategies reveals several key findings. Only five health systems explicitly define a clear vision for their eHealth strategy, while all systems outline objectives. A description of follow-up and evaluation is included in most of the strategies, though the level of detail varies across the systems. Regarding the working methods, coordination with other national strategies, and stakeholder cooperation in implementation, there is a noticeable variation among the strategies, with some systems providing more detailed descriptions than others. This diversity highlights different approaches and priorities in the development and implementation of eHealth strategies across the studied health systems (See Table 3 for an overview of the Results).

**Table 3:** Summary of findings in the different strategies and associated documents

	**Australia**	**Catalonia**	**Denmark**	**Estonia**	**Finland**	**Norway**	**Sweden**	**NHS England**	**Veterans Affairs**
**Visions and objectives**									
eHealth Vision	Yes	No	No	Yes	Yes	Yes	Yes	No	No
Focus of strategy	Citizen Health	Health	Health	Health	Health	Health	Country IT	Health	Organi- zation IT
Strategic Areas	PI, TM, PH, IM, ST, CT, DA	PI, TM, IM, ST, CT, DA	PI, TM, PH, IM, ST, CT	PI, TM, PH, IM, ST, CT, DA	PI, TM, PH, IM, ST, CT, DA	PI, TM, PH, IM, ST, CT, DA	PI, IM, ST, CT	PI, TM, IM, ST, DA	PI, TM, PH, IM, ST, CT, DA
**Means to reach the objectives**									
Development process									
Multiple stakeholder involvement	Yes	Yes	Yes	Yes	Yes	Yes	No	Yes	Yes
Public process	Yes	Yes	Yes	Yes	Yes	Yes	No	No	No
Relation to other strategies	Described	vaguely described	Not described	Described	Described	Described	Not described	vaguely described	Described
Coordination of eHealth strategy	Described	Described	Described	Described	Described	Described	Described	vaguely described	Described
Working methods	Described	Not described	Not described	Described	Not described	Not described	Not described	Described	Not described
Stakeholder cooperation	Described	Described	vaguely described	Described	vaguely described	Described	vaguely described	Described	vaguely described
** Structures for follow up**									
Structures for follow up	Described	Not described	Described	Described	Described	Described	Not described	Not described	Not described

*Abbreviations: PI* Patient Involvement, *TM* Telemedicine, *PH* Preventive Healthcare, *IM* Information Management, *ST* Standards, *CT* Consistent use of Terms, *DA* Data Analysis

## Discussion

This study aimed to compare the commonalities and differences across nine health systems in terms of eHealth vision and objectives, methods for achieving these objectives, and structures for follow-up.

The findings reveal that five of the studied health systems express a clear eHealth vision. However, their focuses differ: some emphasize technical development (e.g., Australia), organizational coherence (e.g., Finland), or national excellence (e.g., Sweden). While most place the patient at the center of their vision, this is not universal. The presence of clear eHealth visions in five systems is advantageous, as prior research suggests that a strong organizational vision can guide decision-making, align stakeholders, and improve overall performance [33]. Many existing studies on health and eHealth strategies have not explicitly linked strategic goals to objectives [34–39]. In contrast, our study finds that Sweden’s strategy stands out by covering only four strategic goals, compared to six or seven in most of the other studied systems. Furthermore, all strategies, except those from NHS England and Sweden, incorporate eHealth within the context of preventive health.

Regarding the means to achieve objectives, the weakest aspect across the analyzed systems appears to be the description of the eHealth strategy development process. Hudson et al. [40] stress the importance of collaboration and stakeholder involvement in eHealth development and implementation. This is further emphasized by the WHO, which, in its 2012 strategy toolkit, highlighted the value of engaging a wide range of stakeholders early on to lend credibility to the strategy and incorporate valuable expertise [17]. Engaging stakeholders early increases the likelihood of having sufficient resources, power, and capabilities to successfully execute the strategy [41]. Systems like Australia, Estonia, Finland, and Norway, which involve a broader range of stakeholders, may have a better chance of success compared to Sweden, where the strategy was developed without key stakeholder input. The study also finds that some systems explicitly integrate their eHealth strategies with other national strategies (e.g., Australia, Finland), while others make this connection less clear (e.g., Estonia, NHS England). Some systems, such as Norway and Sweden, keep the eHealth strategy isolated. Without clear integration with other national strategies, there is a risk that the planning process will lack an overarching perspective, which can potentially lead to conflicting strategies [24].

Existing research typically does not examine the means and actors responsible for implementing eHealth strategies [34–39]. Our findings indicate that many systems do not clearly define how implementation will be carried out, even though they identify responsible actors. One system (the VA) provides no details at all, and only three systems (Australia, Estonia, and NHS England) provide specifics about both the actors involved and the implementation process. To determine whether a strategy has achieved its intended outcomes, it is essential to measure and evaluate these results [42, 43]. While all the studied systems mention follow-up in some capacity, Catalonia, Sweden, NHS England, and the VA lack explicit follow-up measures. Norway provides a useful example of follow-up in their Plan for the implementation of the National e-Health Strategy where they define Name of indicator, Unit of measurement, Type of indicator, Description and purpose, How measurements shall be carried out, Target value (to reach in 2025), and starting point (as of 2023).

### Implications for practice

As a decision-maker with influence over the creation or development of eHealth strategies, analyzing the approaches of other health systems can provide valuable insights and inspiration. However, it is important to exercise caution when drawing inspiration from existing strategies, as eHealth strategies differ significantly across systems in terms of focus, scope, and implementation. Based on the findings in this study, it can be concluded that Australia’s and Estonia’s eHealth strategies are among the most comprehensive and well-structured to date. In addition, Norway’s Plan for implementation is detailed in its description of activities and evaluation. This makes them particularly valuable models for other countries or regions looking to design or improve their own eHealth frameworks.

Australia’s eHealth strategy stands out for its, in comparison, well-described development process together with description of partners and governance. The strategy is comprehensive in its integration with broader national health and digitalization goals, ensuring alignment with other national policies. In addition, the practical measures taken in Australia to measure performance will be based on the Quintuple Aim for Healthcare Improvement [44]. This approach reflects a strong commitment to both innovation and evidence-based practice, ensuring that the implemented solutions are effective and scalable.

Estonia is another example of a well-structured and forward-thinking eHealth strategy. Estonia’s approach is characterized by a high level of integration with the country’s digital infrastructure, as well as a strong emphasis on digital health literacy and patient-centered care. The strategy also prioritizes innovation, cybersecurity, and the efficient use of health data. The Estonian strategy’s focus on strong governance, clear objectives, and ongoing evaluation also contributes to its effectiveness.

Drawing inspiration from Australia and Estonia, decision-makers should prioritize clear vision statements that align with both national and health-specific objectives. Additionally, ensuring stakeholder involvement from the outset is crucial. This can increase the chances of successful strategy development and help identify potential barriers early on. Another valuable takeaway is the importance of structured follow-up and evaluation mechanisms, which are critical for ensuring the sustainability and impact of eHealth initiatives over time.

However, while these two strategies provide strong examples, it is essential to recognize that no single strategy will work universally across all contexts. Decision-makers must consider their own national or regional healthcare needs, technological infrastructure, and societal priorities when adapting or implementing these models.

### Limitations

While this study provides valuable insights into eHealth policies across nine health systems, several limitations must be acknowledged. First, the study was limited to systems meeting specific inclusion criteria, such as a well-established healthcare framework and the availability of eHealth strategies in certain languages. This inevitably excluded other health systems that may offer valuable perspectives, particularly those from non-OECD countries or systems with differing healthcare funding mechanisms. Moreover, the linguistic focus on strategies published in English, Swedish, Danish, or Norwegian may have further narrowed the scope, leaving out significant contributions from regions where documentation is unavailable in these languages.

The emphasis on health systems with comparable funding and coverage structures—specifically single-payer, tax-funded models with universal healthcare coverage—ensured consistency in the comparative analysis. However, it inherently excluded systems operating under different organizational models, such as multi-payer systems or those without universal coverage, which could have provided insights shaped by distinct challenges and priorities. Additionally, the reliance on publicly available strategy documents means that certain aspects, such as the nuances of the development process, stakeholder dynamics, or practical implementation challenges, may not have been fully captured. Internal documentation or interviews with stakeholders could have offered a more comprehensive understanding of the strategies analyzed.

The study also centered on three key dimensions: vision and objectives, means to achieve objectives, and structures for follow-up. While these are important areas for evaluating eHealth strategies, other critical factors—such as the socio-political context, levels of financial investment, and infrastructure readiness—were not explored, leaving gaps in the overall analysis. Furthermore, the study did not assess the real-world effectiveness of the strategies, such as their impact on healthcare delivery, patient outcomes, or system efficiency. By focusing solely on document-based analysis, the findings do not address whether these strategies achieved their intended goals or yielded tangible benefits in practice.

### Future research

To deepen understanding and further explore the topic of eHealth strategies, future research could focus on several key areas. First, expanding the scope to include non-OECD countries and health systems with alternative funding mechanisms, such as multi-payer systems or those without universal healthcare coverage, could provide valuable insights into how varying socio-economic and organizational contexts influence the development and implementation of eHealth strategies. Such comparative analyses would enhance the understanding of global diversity in eHealth approaches. Second, examining the practical application of eHealth strategies is essential. This includes studying their impacts on healthcare delivery, patient outcomes, and system efficiency. Mixed-method approaches, combining qualitative document analysis with quantitative outcome assessments, could offer a comprehensive view of their effectiveness. Third, qualitative research involving interviews with stakeholders, including policymakers, healthcare professionals, and patients, could reveal the roles, experiences, and contributions of these groups in the creation and execution of eHealth strategies. This approach would help identify best practices for fostering collaboration and managing conflicts across diverse contexts. Finally, exploring the influence of socio-political environments on eHealth strategy formulation and execution is a promising avenue. This could involve analyzing governance structures, regulatory frameworks, and cultural factors. Cross-country comparisons in this domain would provide insights into how different socio-political contexts shape strategic priorities and implementation methods.

## Conclusion

This study provides a comparative analysis of nine eHealth strategies, highlighting their vision and objectives, means to achieve these objectives, and structures for follow-up. The findings demonstrate considerable variability in the comprehensiveness and focus of these strategies, reflecting differences in socio-economic contexts, healthcare system structures, and strategic priorities. While five systems articulate a clear vision with varied emphases such as technical development, organizational coherence, or national excellence, others exhibit gaps in strategic goals or connections to preventive health. The analysis underscores the importance of stakeholder engagement and integration with broader policy frameworks in the development and implementation of eHealth strategies. Strategies like Australia’s, Estonia’s and Norway’s exemplify more detailed and cohesive approaches, emphasizing stakeholder involvement and alignment with other governing strategies, but also the importance of a pre-defined follow up and evaluation. However, many systems lack clarity in describing implementation plans or explicit measures for follow-up, leaving room for improvement in ensuring strategy execution and accountability.

The study also highlights limitations, such as the exclusion of non-OECD countries and systems with diverse funding mechanisms, reliance on publicly available documents, and a lack of evaluation of real-world effectiveness. These limitations point to opportunities for future research to broaden the scope, assess practical impacts, and explore socio-political influences on eHealth strategy development. In conclusion, while this study offers valuable insights into the commonalities and differences among eHealth strategies, it also underscores the need for tailored approaches that account for specific healthcare contexts. Policymakers can draw inspiration from the more comprehensive strategies analyzed but must adapt these lessons to their unique challenges and goals. Through iterative refinement, broader inclusion, and detailed follow-up processes, eHealth strategies can play a pivotal role in advancing healthcare systems worldwide.

## Supplementary Information

Supplementary Material 1.

Supplementary Material 2.

## Data Availability

Data is provided in supplementary information file, Appendix 2.
