# Supplementary material for: National eHealth strategies: a comparative study of nine OECD health systems
Source: BMC Health Serv Res. 2025 Feb 18;25:269. doi: 10.1186/s12913-025-12411-7 (PMC11834240; doi:10.1186/s12913-025-12411-7)
Supplement: Supplementary file 1 — Supplementary Material 1. [file 12913_2025_12411_MOESM1_ESM.docx]

**Appendix 1**

**Further information on different countries’ health systems, organized by country**

**The United Kingdom**

1. Anderson M, Pitchforth E, Edwards N, Alderwick H, McGuire A, Mossialos E. The United Kingdom: Health system review. Health Systems in Transition, 2022; 24(1): i–192. ISSN 1817-6119

**Spain**

1. Bernal-Delgado E, Angulo-Pueyo E, Ridao-López M, Urbanos-Garrido RM, Oliva-Moreno J, García-Abiétar D, Hernández-Quevedo C. Spain: Health system review. Health Systems in Transition. Copenhagen: WHO Regional Office for Europe; 2024, 26(3): i-187. ISBN 978 9 2890 5970 1

**Denmark**

1. Birk HO, Vrangbæk K, Rudkjøbing A, Krasnik A, Eriksen A, Richardson E, Smith Jervelund S. Denmark: Health system review. Health Systems in Transition, 2024; 26(1): i–152. ISBN 978 92 890 5956 5

**Australia**

1. Dixit SK, Sambasivan M. A review of the Australian healthcare system: A policy perspective. SAGE Open Med. 2018 Apr 12;6:2050312118769211. doi: 10.1177/2050312118769211. PMID: 29686869; PMCID: PMC5900819.

**Sweden**

1. Janlöv N, Blume S, Glenngård AH, Hanspers K, Anell A, Merkur S. Sweden: Health system review. Health Systems in Transition, 2023; 25(4): i–198. ISBN 978 9 2890 5943 5

**Estonia**

1. Kasekamp K, Habicht T, Võrk A, Köhler K, Reinap M, Kahur K, Laarmann H, Litvinova Y. Estonia: Health system review. Health Systems in Transition, 2023; 25(5): i–204. ISBN 9789289059510

**Finland**

1. Keskimäki I, Tynkkynen LK, Reissell E, Koivusalo M, Syrjä V, Vuorenkoski L, Rechel B, Karanikolos M. Finland: Health system review. Health Systems in Transition, 2019; 21(2): 1 – 166 ISSN 1817-6119

**USA**

1. Rice T, Rosenau P, Unruh LY, Barnes AJ, van Ginneken E. United States of America: Health system review. Health Systems in Transition, 2020; 22(4): pp.i–441. ISSN 1817-6127

**Norway**

1. Saunes I S, Karanikolos M, Sagan A. Norway: Health system review. Health systems in Transition, 2020; 22(1): i–163. ISSN 1817-6119
