# Supplementary material for: National eHealth strategies: a comparative study of nine OECD health systems
Source: BMC Health Serv Res. 2025 Feb 18;25:269. doi: 10.1186/s12913-025-12411-7 (PMC11834240; doi:10.1186/s12913-025-12411-7)
Supplement: Supplementary file 2 — Supplementary Material 2. [file 12913_2025_12411_MOESM2_ESM.docx]

**Appendix 2**

**Documents used in the study, organized based on health systems**

**Australia**

1. Australian Digital Health Agency. 2023. National Digital Health Strategy 2023-2028. ISBN 978-0-6459861-0-5
2. Australian Digital Health Agency. 2023. National Digital Health Strategy Delivery Roadmap 2023-2028. ISBN 978-0-6459861-1-2

**Catalonia**

1. Ministry of Health. 2017. The Catalan Information Systems Master Plan. Building a digital health strategy for Catalonia together.

**Denmark**

1. Regeringen. Finansministeriet. 2022. Danmarks digitaliserings strategi Sammen om den digitale udvikling. ISBN 978-87-94224-16-1
2. Danish Ministry of Health Danish Ministry of Finance Danish Regions Local Government Denmark. 2018. - A Coherent and Trustworthy Health Network for All; Digital Health Strategy 2018-2022
3. Danske Regioner, Sundhedsdatastyrelsen. 2019. Introduktion til indikatorer for udbredelse og anvendelse af sundheds-it

**Estonia**

1. eHealth task force. 2015. Estonian eHealth Strategic Development Plan 2020.
2. Press release Government. 2014-07-03. [Online]
   https://riigikantselei.ee/en/news/task-force-be-created-will-begin-resolve-ehealth-problems.

**Finland**

1. Ministry of Social Affairs and Health. 2024:1. Strategy for digitalization and information management in healthcare and social welfare. ISBN 978-952-00-5404-5
2. Finnish Institute for Health and Welfare. 2022. Report 6. E-health and e-welfare of Finland Check Point 2022.

**Norway**

1. Direktoratet for e-helse. 2023. Nasjonal e-helsestrategi
2. Direktoratet for e-helse. 2021. Plan for internasjonale standarder 2021-2024. IE-1079
3. Direktoratet for e-helse. Mandat for Nasjonalt e-helseråd (E-helserådet).
4. Direktoratet for e-helse. Mandat for Prioriteringsutvalget (NUIT).
5. Direktoratet for e-helse. Mandat for Fagutvalget (NUFA).
6. Direktoratet for e-helse. 2023. Nasjonal e-helseportefølje februar 2023. Versjon 1.0. IE-1115
7. Direktoratet for e-helse. 2023. Plan for realisering av Nasjonal e‑helsestrategi. Versjon 0.95. IE-1113

**Sweden**

1. Ministry of Health and Social Affairs. Swedish Association of Local Authorities and Regions. 2016. Vision for eHealth 2025 – common starting points for digitisation of social services and health care. S2016/01874/FS
2. Ministry of Health and Social Affairs. Swedish Association of Local Authorities and Regions. 2020. A strategy for implementing Vision for eHealth 2025. The next step
3. Implementation plan 2020–2022. Appendix to Strategy document Vision e-health 2025. Version May 2022. In Swedish.
4. eHealth Agency. 2023. Follow-up Vision for eHealth 2025. Report on the Year 2022. Rfn. 2021/00171

**NHS England**

1. NHS. 2019. The NHS Long Term Plan. Version 1.2 with corrections.

**Veterans Affairs**

1. U.S. Department of Veterans Affairs. 2018. Department of Veterans Affairs FY 2018 - 2024 Strategic Plan. Section 508 Compliance Statement
2. U.S. Department of Veterans Affairs. Office of Information and Technology. Digital Transformation. It Starts With Us. Information Resource Management FY2020–2022 IT (IRM) Strategic Plan.
3. U.S. Department of Veterans Affairs. Office of Information and Technology. 2020. Digital Transformation. VA Enterprise Roadmap. FY 2020–2026.
